# Nanoscale Three-Dimensional Imaging of Integrated Circuits Using a Scanning Electron Microscope and Transition-Edge Sensor Spectrometer

**DOI:** 10.3390/s24092890

**Published:** 2024-04-30

**Authors:** Nathan Nakamura, Paul Szypryt, Amber L. Dagel, Bradley K. Alpert, Douglas A. Bennett, William Bertrand Doriese, Malcolm Durkin, Joseph W. Fowler, Dylan T. Fox, Johnathon D. Gard, Ryan N. Goodner, James Zachariah Harris, Gene C. Hilton, Edward S. Jimenez, Burke L. Kernen, Kurt W. Larson, Zachary H. Levine, Daniel McArthur, Kelsey M. Morgan, Galen C. O’Neil, Nathan J. Ortiz, Christine G. Pappas, Carl D. Reintsema, Daniel R. Schmidt, Peter A. Schultz, Kyle R. Thompson, Joel N. Ullom, Leila Vale, Courtenay T. Vaughan, Christopher Walker, Joel C. Weber, Jason W. Wheeler, Daniel S. Swetz

**Affiliations:** 1 National Institute of Standards and Technology, Boulder, CO 80305, USA; paul.szypryt@nist.gov (P.S.); bradley.alpert@nist.gov (B.K.A.); douglas.bennett@nist.gov (D.A.B.); william.doriese@nist.gov (W.B.D.); malcolm.durkin@nist.gov (M.D.); joe.fowler@nist.gov (J.W.F.); johnathon.gard@colorado.edu (J.D.G.); kelsey.morgan@nist.gov (K.M.M.); galen.oneil@nist.gov (G.C.O.); nathan.ortiz@nist.gov (N.J.O.); carl.reintsema@nist.gov (C.D.R.); dan.schmidt@nist.gov (D.R.S.); joel.ullom@nist.gov (J.N.U.); leila.vale@nist.gov (L.V.); joel.weber@nist.gov (J.C.W.); daniel.swetz@nist.gov (D.S.S.); 2 Department of Physics, University of Colorado, Boulder, CO 80309, USA; 3 Sandia National Laboratories, Albuquerque, NM 87123, USA; aldagel@sandia.gov (A.L.D.); dylfox@sandia.gov (D.T.F.); rngoodn@sandia.gov (R.N.G.); esjimen@sandia.gov (E.S.J.); blkerne@sandia.gov (B.L.K.); dmcarth@sandia.gov (D.M.); paschul@sandia.gov (P.A.S.); krthomp@sandia.gov (K.R.T.); ctvaugh@sandia.gov (C.T.V.); cwalke@sandia.gov (C.W.); jwwheel@sandia.gov (J.W.W.); 4 National Institute of Standards and Technology, Gaithersburg, MD 20899, USA; zachary.levine@nist.gov

**Keywords:** X-ray nanotomography, X-ray imaging, integrated circuits, computed tomography

## Abstract

X-ray nanotomography is a powerful tool for the characterization of nanoscale materials and structures, but it is difficult to implement due to the competing requirements of X-ray flux and spot size. Due to this constraint, state-of-the-art nanotomography is predominantly performed at large synchrotron facilities. We present a laboratory-scale nanotomography instrument that achieves nanoscale spatial resolution while addressing the limitations of conventional tomography tools. The instrument combines the electron beam of a scanning electron microscope (SEM) with the precise, broadband X-ray detection of a superconducting transition-edge sensor (TES) microcalorimeter. The electron beam generates a highly focused X-ray spot on a metal target held micrometers away from the sample of interest, while the TES spectrometer isolates target photons with a high signal-to-noise ratio. This combination of a focused X-ray spot, energy-resolved X-ray detection, and unique system geometry enables nanoscale, element-specific X-ray imaging in a compact footprint. The proof of concept for this approach to X-ray nanotomography is demonstrated by imaging 160 nm features in three dimensions in six layers of a Cu-SiO_2_ integrated circuit, and a path toward finer resolution and enhanced imaging capabilities is discussed.

## 1. Introduction

Nanoscale three-dimensional X-ray imaging yields insight into the internal structure of materials that would otherwise be difficult to probe, with applications spanning many emerging technologies such as advanced energy storage materials [1] and next-generation integrated circuits (ICs) [2]. Modern nanoelectronics, in particular, would benefit from advanced nanoscale imaging, with recent reports highlighting nanoscale resolution and sensitivity to material composition as potentially transformative capabilities for nanoelectronics characterization [3]. X-ray absorption computed tomography (CT) is a well-developed method for three-dimensional imaging, relying on differences in X-ray transmission to determine the size and shape of subsurface features [4]. A CT scan consists of acquiring many two-dimensional radiographs of a sample at different projection angles and combining these projections into a three-dimensional reconstructed image. In general, the X-ray focal spot size must be comparable to the desired spatial resolution when collecting each radiograph. This requirement limits the number of available photons when pushing the spatial resolution to the order of tens to hundreds of nanometers, leading to long collection times and low signal-to-noise ratios. For this reason, X-ray nanotomography has largely been deployed at synchrotron beamlines, where a high X-ray flux can be maintained even with nanoscale X-ray spot sizes [5,6,7].

A need exists for more accessible and disseminable nanotomography systems, capable of resolving buried nanoscale features in modern ICs within a compact instrument footprint. Despite this need, current tomography instruments do not provide the capabilities required for nanoscale IC characterization. Current laboratory-scale tools capable of achieving the best spatial resolution rely on X-ray optics and destructive sample preparation techniques [8,9]. The highest spatial resolution is achieved at lower X-ray energies, which leads to high attenuation and low detected X-ray flux in thicker samples or high-Z components. This results in the majority of laboratory nanotomography instruments destroying the sample outside of a pre-selected micrometer-scale region, severely limiting the available scan area. This is a key limiting factor in nanoelectronics inspection, as the location of compromised features is often unknown prior to imaging, and thus the region of interest cannot be reliably chosen prior to performing tomography. Another approach to achieving high-resolution X-ray CT in a compact footprint is to utilize the electron beam from a scanning electron microscope (SEM) and a metal target as the X-ray source. A small number of SEM-based systems have been developed [10,11,12,13], but none meet the spatial resolution requirements to characterize features smaller than 200 nm. Additionally, current tools characterize only the size and shape of sample features and provide limited information regarding the elemental composition. Alternative approaches utilizing novel X-ray target design, X-ray detection, and system geometry are still required to improve the achievable spatial resolution and imaging capabilities of laboratory CT systems.

**Figure 1:**
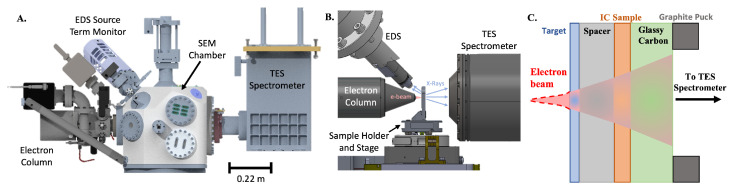
(**A**) MINT overview, consisting of an electron column, energy-dispersive spectroscopy (EDS) source-term monitor, SEM chamber, and TES spectrometer. (**B**) View inside the SEM chamber, showing the electron beam incident on a sample in the sample holder and the generated X-rays going to the TES and EDS. (**C**) Schematic demonstrating the MINT sample configuration and X-ray generation in sample layers: an electron beam incident on a target layer generates X-rays in a nanoscale spot size, which are attenuated by the IC and detected by the TES. Electrons not stopped in the target layer spread into a larger spot size and generate X-rays in other layers of the sample. Sample thicknesses are not drawn to scale. Part B is reprinted from Ref. [18] with permission (http://creativecommons.org/licenses/by/4.0/, accessed on 10 October 2023).

Here, we demonstrate MINT (Microscope for Integrated circuit NanoTomography), a compact X-ray CT system capable of achieving nanoscale spatial resolution and elemental sensitivity in three dimensions. MINT is governed by a different set of limitations from conventional approaches to X-ray nanotomography, allowing it to image nanoscale features without the use of X-ray focusing optics while preserving large-area planar samples, and providing information regarding the elemental composition of samples. MINT combines the electron beam of a scanning electron microscope (SEM) with a transition-edge sensor (TES) X-ray spectrometer (Figure 1A,B). A highly focused electron beam incident on a thin-film metal target generates X-rays, which are attenuated by the sample and detected by the TES spectrometer (Figure 1C). This results in an instrument with a nanoscale X-ray spot size, efficient and energy-resolved X-ray detection, and a system geometry enabling high magnification. Electron beam spots can routinely be focused to the nanometer scale, enabling a nanoscale X-ray generation volume within the thin-film target. The X-rays generated in this nanoscale spot consist of both characteristic fluorescence lines at specific energy levels and a broadband bremsstrahlung background. Additional bremsstrahlung will be generated from other layers in the sample stack with a more diffuse spot size due to electrons that are not stopped in the target and produce X-rays in subsequent layers. To maintain nanoscale spatial resolution, only photons generated in the nanoscale spot size in the metal target layer should be used for tomographic reconstruction. Therefore, an energy-resolving X-ray detector is required to distinguish characteristic target X-rays from background photons generated elsewhere in the sample. MINT utilizes a TES spectrometer for X-ray detection, which consists of hundreds of superconducting microcalorimeter TES pixels [14]. A TES microcalorimeter pixel is an efficient single-photon counting detector operated at cryogenic temperatures capable of extremely high energy-resolving power and detection across a broadband energy range with high precision [15]. Each TES pixel can thus isolate characteristic X-rays generated in the target layer with a high signal-to-noise ratio, ensuring that only X-rays generated in the nanoscale target generation volume are used for tomographic reconstruction. Energy-resolved detection also enables energy binning in reconstructions, which can enable elemental specificity and improve the final image contrast [16,17]. The use of a thin-film metal target also allows the target layer to be deposited directly onto the sample, which generates the X-ray source within micrometers of the sample. This allows for high geometric magnification while keeping the detector relatively close to the source, improving the solid angle coverage and X-ray flux at the detector plane. This combination of a highly focused electron beam, thin-film metal target, efficient energy-resolved X-ray detection, and high geometric magnification enables nanoscale three-dimensional imaging within a compact instrument. The total footprint of MINT is 1.65 m × 1.30 m, and it is 2.00 m tall at its highest point, compact enough to operate in most standard laboratory spaces. This approach to laboratory X-ray tomography provides an alternative method for nanoscale imaging with a promising path forward.

MINT was developed with nanoelectronics as the application space, and so an IC consisting of Cu wiring in a SiO_2_ dielectric fabricated at the 130 nm technology node is used to demonstrate the imaging capability. ICs are a critical use case for nanotomography, as modern ICs are comprised of nanoscale features and many fabrication layers. This structural complexity makes precise imaging and characterization of subsurface layers difficult, limiting critical IC diagnostics such as defect detection and failure analysis [2,19]. ICs pose an additional challenge for traditional sample preparation, as their planar geometry either inhibits rotation or requires that all but a small subsection of the IC be destroyed. This eliminates the ability to scan multiple regions of a sample and limits the ability to use the same sample for complementary diagnostic measurements. The source, sample, and system geometry, along with a custom tomographic reconstruction algorithm, allow MINT to overcome this challenge and keep planar samples intact during imaging.

MINT is the first implementation of a TES X-ray spectrometer for three-dimensional CT imaging and represents a proof of concept for the combination of an SEM, TES, and the novel sample and system geometry presented here. The current instrument is photon-limited, and it is expected that higher-efficiency X-ray detection or higher pixel-count TES spectrometers will be required to achieve faster imaging speeds. Our prior work has described the development of a higher pixel-count spectrometer for three-dimensional imaging applications, but the development of this spectrometer is outside the scope of the current work [20]. Prior work has also described the tomographic reconstruction approach required to generate three-dimensional images from MINT data but does not provide details on the system physics or instrumental approach to enable laboratory-nanoscale CT of integrated circuits [21]. Here, the first description of the instrument design and underlying physics necessary to achieve nanoscale three-dimensional X-ray imaging with MINT is presented, providing a roadmap for nanoscale X-ray CT in an SEM-based tabletop instrument. In MINT, the electron beam conditions, X-ray target materials and dimensions, system magnification, and energy of photons used in the reconstruction can be tuned for optimized imaging of a wide variety of samples and material compositions. The imaging demonstration discussed here is comparable to current state-of-the-art laboratory-scale CT instruments in terms of the achievable spatial resolution on IC samples, with additional elemental detection capabilities not found elsewhere. The unique source and system setup of MINT lead to a different set of limiting factors compared to conventional approaches to laboratory CT, and a clear path forward exists to improve the imaging speed, spatial resolution, and spectral imaging capabilities.

## 2. MINT System Description

### 2.1. X-ray Source

The X-ray source has a significant influence on the final image quality. The focal spot size of the X-ray source impacts the achieved spatial resolution [22,23], and attenuation in the sample is a function of the incident X-ray energy as well as the size, shape, and composition of materials. In MINT, the X-ray source is carefully optimized by adjusting the target and electron beam parameters to maximize the imaging speed for a given sample. Ideally, the source would maximize the X-ray flux at X-ray energies that provide high imaging contrast for a given sample while maintaining a spot size near the desired spatial resolution. As the IC sample used here was fabricated at the 130 nm node, we aimed to maintain a focal spot size at or below 130 nm.

#### 2.1.1. Target Selection

The thin-film target thickness and material can be selected to maximize the imaging speed for a given sample and desired spatial resolution. The final imaging speed depends on the electron-stopping power of the target material and the energy of the characteristic X-ray lines emitted. Thicker, higher *Z* targets stop more electrons and thus generate more X-ray flux but also result in larger X-ray focal spot sizes. The energy of the emitted fluorescence line influences the expected contrast, which we define as the difference in attenuation between Cu and SiO_2_ at a given X-ray energy (Δμ). Thus, a target material should be chosen to maximize X-ray generation at an energy of high contrast for the sample of interest while maintaining a suitably small X-ray spot size for nanoscale spatial resolution.

A target was selected for the sample IC by developing an estimate of the relative imaging speed based on prospective target materials and thicknesses, as well as the available operating conditions of the SEM electron beam. Prospective target materials were chosen to cover a range of *Z*, with fluorescence lines within the dynamic range of the TES spectrometer and based on fabrication capabilities. The imaging speed estimate is simplified by supposing that we count photons over an energy interval of width ΔE, approximately equal to the TES energy resolution, from which an expected background must be subtracted. The final prediction for the relative imaging speed is given as

(1) $$\frac{1}{t}<f{\left(\Delta \mu \right)}^{2}{\left(1+2b\Delta E/f\right)}^{-1}.$$

where *f* is the fluorescence photon rate, *b* is the bremsstrahlung rate, and 1/t is the imaging speed. A full derivation of Equation (1) and a method to predict the absolute imaging speed can be found in the Supplementary Materials, Section S1. The absolute imaging speed includes the voxel size, which is related to the achievable spatial resolution of the system. The goal of the imaging speed estimate here is to assess the relative performance of certain target materials and thicknesses at a constant voxel size; thus, the voxel size was omitted from Equation (1). The first two factors show that the speed scales with the fluorescence photon rate *f* and with the square of the material absorption contrast. The last factor represents a slowdown due to subtracting background from the observed counts.

The photon yields *f* and *b* were predicted using the Monte Carlo electron-photon transport code PENELOPE (Penetration and ENErgy LOss of Positrons and Electrons), version 2018 [24]. The library was driven via the main program PENEPMA [25]. All PENELOPE results were scaled for the TES spectrometer collection efficiency and solid angle, and for 10 nA of the electron beam current. A 150 nm voxel size and a 100 nm full-width half-maximum (FWHM) Gaussian electron beam spot were assumed. The true electron beam’s FWHM was later characterized to be slightly narrower than the beam size used in PENELOPE (see Section II.A.2). The 100 nm FWHM provides a margin for error in the target thickness selection and ensures that the X-ray spot size remains well below the desired 130 nm FWHM threshold. Target materials were chosen based on which target material and thickness yielded the fastest estimated imaging speed without exceeding the desired maximum X-ray spot size.

**Figure 2:**
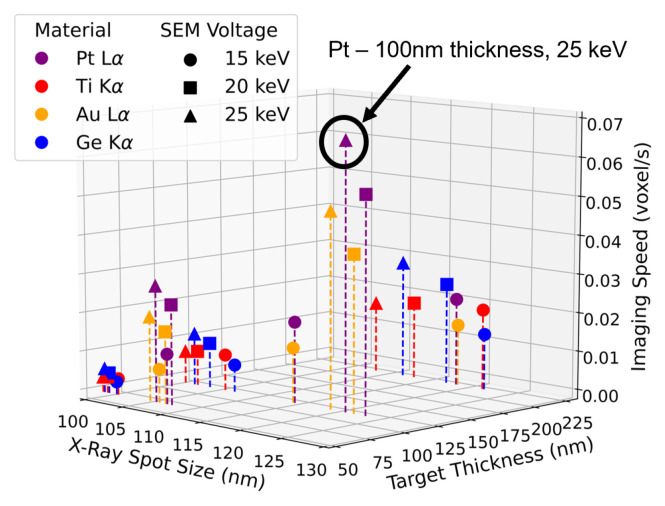
PENELOPE simulation results for the candidate target materials, showing the predicted imaging speed for the coadded TES array. A 100 nm thick Pt layer at an electron accelerating voltage of 25 keV yields the best imaging speed on the selected IC and was thus chosen for the MINT imaging demonstration.

Figure 2 shows the predicted imaging speed versus the X-ray spot FWHM and target thickness for four candidate target materials at three candidate accelerating voltages. Target thicknesses or materials that exceeded the set 130 nm spot size threshold were not included. Candidate materials were selected based on fabrication capabilities and to cover a suitably wide range of X-ray fluorescence energies so as to make an informed decision about the target material. A 100 nm thick Pt target at a 25 keV accelerating voltage yielded the fastest predicted imaging speed and was chosen as the target material for this demonstration. In general, higher electron accelerating voltages produce a smaller volume of X-ray generation, allowing for thicker targets while maintaining a smaller spot size. At these higher accelerating voltages, higher *Z* materials generate X-rays more efficiently. Additionally, the Pt L_*α*_ line is just above the Cu K-edge, leading to a high Cu-SiO_2_ attenuation difference. The Pt L_*α*_ line is also at an energy (9.4 keV) of high TES detection efficiency, improving the number of detected photons. Lastly, higher-energy fluorescence lines transmit through the silicon and glassy carbon layers with less attenuation, leading to more detected photons at the TES spectrometer. The combination of these factors makes the 100 nm Pt target a suitable target for the current imaging demonstration. This target selection process can be repeated for any sample composition or desired imaging spatial resolution, allowing for flexible target design to image samples with varying compositions or feature sizes. The achievable spatial resolution in a given sample depends on a variety of factors, including the spot size, spot shape, and imaging contrast. A complete prediction of the achievable spatial resolution in X-ray tomography systems is outside the scope of the current manuscript. However, in general, spatial resolution can be degraded in exchange for faster imaging speed by increasing the beam current of the electron column, as shown in the next section.

#### 2.1.2. Electron Aperture Selection

MINT utilizes a commercial horizontal electron column (Orsay Physics, Fuveau, France) with a ZrO_2_-coated W emitter. The electron column is critical for achieving high spatial resolutions, as it contributes to the source size and stability. The electron beam spot size sets the X-ray focal spot size, with a smaller electron beam on the metal target producing a smaller X-ray generation volume. The electron beam current sets the X-ray flux and thus the imaging speed, with higher beam currents producing more source X-rays.

The electron beam spot size and current are influenced by a variety of factors, including the accelerating voltage, voltages applied to the condenser and objective lenses, and the choice of aperture size. The accelerating voltage was set to 25 keV to maximize the imaging speed when using the Pt target. The condenser lens voltage was pre-selected based on the electron column conditions, and the objective lens voltage was adjusted at each aperture size to focus the electron beam onto the target surface. This leaves the aperture size as the last remaining variable to tune the electron beam spot size and beam current. A tradeoff exists when selecting the aperture size, as smaller aperture sizes correspond to smaller electron beam sizes but also limit the electron beam current. The largest possible aperture without exceeding the desired spot size should be chosen to maximize the electron beam current and thus the imaging speed at the desired spatial resolution.

**Figure 3:**
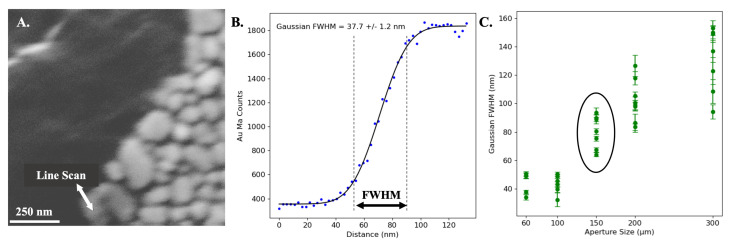
(**A**) The Au-on-C sample. Line scans over the edge of Au grains were collected, and the Au M_*α*_ counts detected by the EDS were extracted. (**B**) Au M_*α*_ counts versus distance along a line scan (blue points) using the 60 μm aperture, fitted to a Gaussian integral (black line) to estimate the electron beam full-width half-maximum (FWHM). (**C**) Estimated Gaussian FWHM versus the SEM aperture size. An aperture size of 150 μm was chosen for tomography in this measurement. At smaller aperture sizes (60 μm and below), the measured spot size becomes limited by the sharpness of the Au edge rather than the size of the electron beam.

To characterize the relationship between the aperture size and electron beam spot size, an Au-on-C calibration sample was used (Ted Pella, Inc., Redding, CA, USA). This sample consists of several Au particles distributed on a C background. The electron beam was scanned perpendicular to the Au edges (Figure 3A), starting on the C background and moving onto the Au particle. Multiple line scans were taken across different Au edges to average out the effect of imperfect edge sharpness on the Au particles. An energy-dispersive spectroscopy (EDS) detector located on the electron beam side of the sample, along with the associated software (Oxford Instruments, v3.3, Concord, MA, USA), was used to select the Au M_*α*_ counts. These data were then fitted to a Gaussian integral to extract the estimated electron beam FWHM (Figure 3B). This process of scanning over a sharp edge has been previously demonstrated as a viable approach to estimating electron beam point-spread functions [26,27,28]. The measurement was repeated across five aperture sizes ranging from 60 μm to 200 μm (Figure 3C). The 150 μm aperture size was chosen for the current demonstration, as it yielded approximately 9 nA of beam current at a Gaussian FWHM of 80 nm. Based on the PENELOPE simulations, the electron beam was expected to spread by 23.6% throughout the 100 nm thick Pt target. This resulted in a predicted X-ray spot size of approximately 100 nm, safely below the desired 130 nm limit.

### 2.2. System Geometry and Magnification

**Figure 4:**
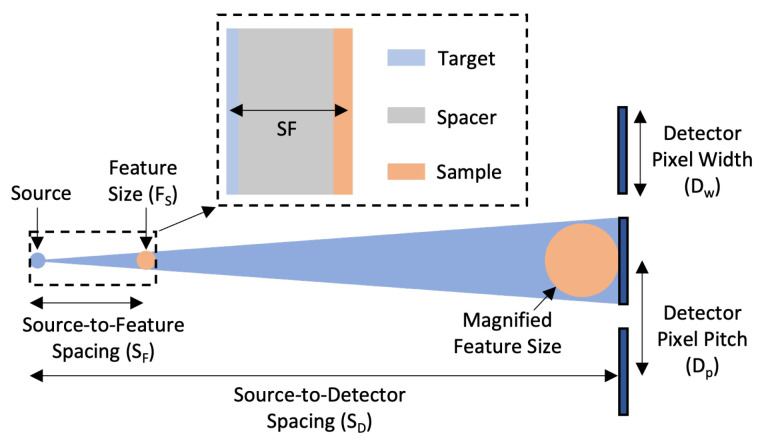
Geometric magnification in MINT. Nanometer-scale features in the IC are magnified onto TES pixels. The design magnification is proportional to the TES pixel pitch ($D_{p}$) and the desired resolvable feature size ($F_{S}$), while the system magnification is proportional to the ratio of the source-to-detector ($S_{D}$) and source-to-feature ($S_{F}$) spacing. The system magnification should be higher than the design magnification to resolve the desired feature size. This figure is reprinted from Ref. [21].

The geometric magnification of an X-ray CT instrument is one factor that determines the achievable spatial resolution (Figure 4). There are two magnification factors to consider: the projection magnification $M_{projection}$ and the system magnification $M_{system}$. $M_{projection}$ is the magnification of a certain feature size onto the X-ray detector and is given by

(2) $$M_{projection}=2\frac{D_{p}}{F_{S}}$$

where $D_{p}$ is the TES pixel pitch and $F_{S}$ is the minimum desired feature size. In the current TES spectrometer, each TES pixel has an aperture 320 μm wide, setting the pixel width. Since pixels in an array do not touch and the pixel pitch (550 μm) is larger than the pixel width, $D_{p}$ must be used to determine the projection magnification. A factor of 2 was included and used to design the imaging geometry to provide a margin for error, as explained further below. The minimum expected feature size in the demonstration IC sample was expected to be 160 nm, leading to a projection magnification of 6875.

To resolve a given feature size, the system magnification should be equal to or exceed the projection magnification. $M_{system}$ is defined as the ratio of the source-to-detector distance ($S_{D}$) and the source-to-feature distance ($S_{F}$)

(3) $$M_{system}=\frac{S_{D}}{S_{F}}$$

High system magnification can be achieved either by moving the detector far from the source or by moving the source close to the sample. In practice, moving the X-ray detector far from the source reduces the solid angle of collection and X-ray flux, leading to increased imaging times and reduced signal-to-noise ratios. In MINT, the detector is moved as close to the source as possible without collisions in the chamber to a distance of 75 mm. High $M_{system}$ is still achieved by depositing the X-ray target directly onto the IC wafer, resulting in a small $S_{F}$ (Supplementary Materials, Section S2). $S_{F}$ is set by the target thickness, the sample thickness, and the thickness of a silicon layer left between the sample and target, referred to as the spacer layer. The thickness of the spacer layer can be tuned to achieve a specific $M_{system}$ based on the desired resolvable feature size and resulting $M_{projection}$. In this demonstration, the spacer was thinned to 8.5 μm, which, with a target thickness of 100 nm and an approximate IC sample thickness of 3.5 μm, resulted in a system magnification of 7300 at normal incidence. This is larger than the projection magnification, indicating that the system geometry of MINT was designed to image features down to at least 160 nm.

$M_{system}$ varies with the angle of incidence and depth in the sample and is used as an estimator to ensure that the system geometry is compatible with the desired spatial resolution. As the angle of incidence θ changes, the source-to-feature distance increases due to the effective thickness of the spacer scaling as secθ. This reduces $M_{system}$ at larger angles. The factor of 2 was included in the design magnification to account for this effect. In general, system parameters such as the TES pixel size and pitch, the source-to-detector distance, and the spacer thickness can be varied to achieve magnification factors suitable for the desired feature size.

### 2.3. TES Spectrometer

**Figure 5:**
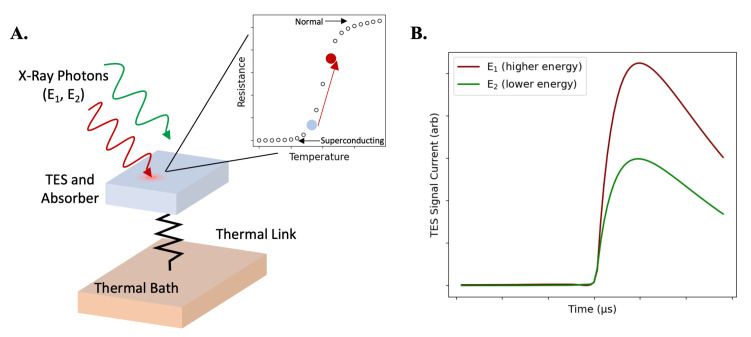
(**A**) Schematic of a TES. The TES and absorber are weakly thermally coupled to a silicon substrate serving as a thermal bath via a silicon nitride membrane. The TES is cooled into its superconducting state and voltage-biased onto the superconducting-to-normal transition. When a photon is absorbed, the small increase in absorber and TES temperature results in a relatively large change in the TES resistance. Higher energy photons cause a larger change in temperature and thus a larger change in the TES resistance. (**B**) The change in TES resistance caused by photon absorption is read out as a negative-going pulse in the TES current (shown inverted here), with the pulse height proportional to the energy of the incident photon.

To isolate X-ray photons generated in the Pt target while still collecting enough photons for imaging, an X-ray detector with both high energy resolution and detection efficiency is required. TES microcalorimeters are an excellent choice for this application, as they bridge the gap between energy-dispersive detectors, which often have high efficiency but poor energy resolution, and wavelength-dispersive detectors, which have high energy resolution but poor efficiency [29]. A TES microcalorimeter is a cryogenic superconducting detector that measures the energy of individual photons with excellent energy resolution [15]. Each MINT TES consists of a 350 μm × 350 μm molybdenum/copper bilayer with eight noise-mitigating copper bars and a critical temperature ($T_{c}$) of 130 mK [30]. A 4.4 μm thick bismuth absorber is deposited on top of each TES to improve X-ray absorption efficiency at higher energies. Each TES is suspended on a silicon nitride (SiN_*x*_) membrane, which serves as a weak thermal link to a silicon substrate. The TES is cooled into a superconducting state, then voltage-biased into the superconducting-to-normal transition regime. When a photon is absorbed, it results in a small change in the temperature of the coupled absorber–TES complex. Due to operating on the steep superconducting-to-normal transition, this small change in temperature results in a relatively large change in the measured TES resistance. The change in resistance from an absorbed photon is observed as a pulse in the measured current, with the pulse height proportional to the energy of the incident photon (Figure 5). TES pixels are often combined into large, multi-pixel arrays to enable higher count-rate measurements while maintaining high energy resolution. The MINT TES array consists of 240 pixels, and they are read out using the time-division multiplexing (TDM) approach [31]. The entire 240-pixel array is cooled to cryogenic temperatures to put the pixels into a superconducting state using a pulse-tube-backed two-stage adiabatic demagnetization refrigerator (ADR) [14,32]. Additional details on TES physics and operation can be found in a number of other publications [15,33].

### 2.4. Source-Term Monitor

The source intensity can fluctuate throughout the measurement due to instabilities in the electron beam current or small variations in the Pt layer thickness at different dwell positions. It is critical to accurately measure this source intensity, as the detected counts must be normalized by the strength of the X-ray source to obtain accurate estimations of the attenuation through the sample. An EDS detector (Oxford Instruments) is located on the target side of the sample and operated continuously during data collection. The EDS detector monitors the total and Pt L_*α*_ count rates during each measurement and is used to quantify the X-ray source intensity.

## 3. Data Processing

### 3.1. TES Data Processing and Analysis

**Figure 6:**
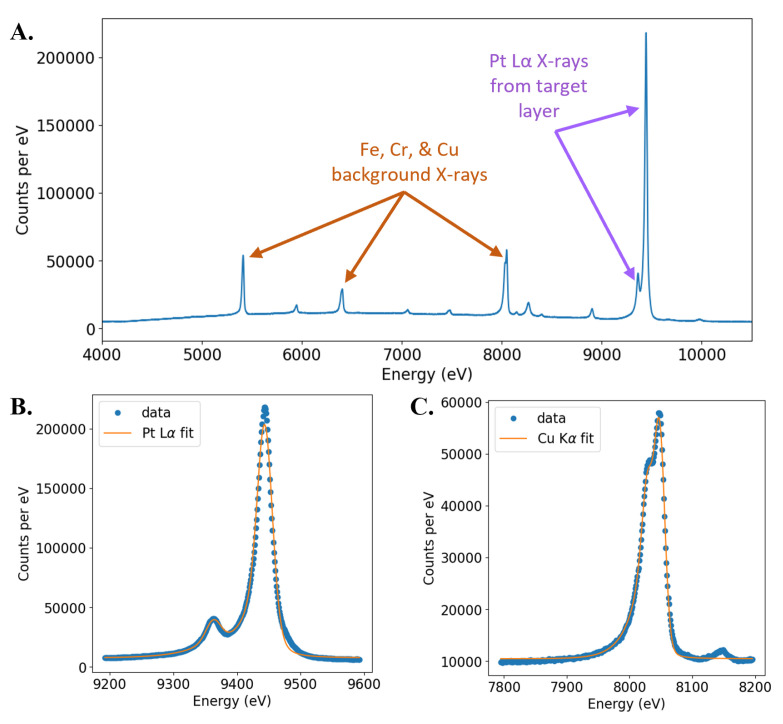
(**A**) Energy-calibrated TES spectrum combined over all TES pixels in the array and over all dwell positions. Purple arrows indicate the Pt L_*α*_ characteristic X-rays used for tomographic reconstruction. All other characteristic X-ray peaks are background peaks from sources such as the SEM chamber, sample holder, or cryostat. A selection of higher-intensity background peaks is indicated by orange arrows. (**B**) Fit to the Pt L_*α*_ line for the full TES array over all dwell positions. This fit separates the Pt X-rays generated in the target layer from the bremsstrahlung background photons. (**C**) Fit to the Cu K_*α*_ line for the full TES array over all dwell positions. The Cu K_*α*_ intrinsic line shape is well characterized, [36,37], and this spectrum was used to establish the energy resolution of the TES spectrometer at 8 keV.

The broadband detection of the TES spectrometer allows for energies from approximately 4–12 keV to be collected simultaneously. The TES spectra can be used to isolate the Pt L_*α*_ fluorescence and select other energy bins of interest for use in tomographic reconstruction. Prior to the collection of CT data each day, a noise record of pulse-free TES current is obtained for each sensor. During CT data collection, each TES pixel in the array collects raw data pulses corresponding to X-ray events incident on the detector. Then, TES X-ray pulse and noise records are reduced to a time-lagged, energy-calibrated spectrum using the Microcalorimeter Analysis Software System (MASS) [34]. Additional details on the pulse processing steps can be found in the Supplementary Materials, Section S3.A.1, as well as in a number of previous publications [34,35]. The output of this processing is an energy-calibrated spectrum spanning the dynamic range of the TES spectrometer (Figure 6A).

An X-ray spectrum is generated for each TES pixel at each dwell position during a tomographic scan, and information from the spectrum is input to a tomographic reconstruction code. Prior to use in reconstruction, each pixel is passed through a series of checks to ensure that lower-quality data are excluded (Supplementary Materials, Section S3.A.2). Extracting Pt L_*α*_ photons to use in the reconstruction can be done in two ways. First, the Pt L_*α*_ line can be fitted to obtain the fluorescence counts at each dwell position for each TES (Figure 6B). The fit consists of two Voigt functions of a known line shape for the Pt L_α_1,2__ doublet [38] with exponential tails to account for the TES detector response function [39]. This isolates only Pt L_α_1,2__ fluorescence and ensures that all photons used for reconstruction were generated in nanoscale spot size. Another method to isolate photons generated in the target layer is to include all energy bins around the Pt L_*α*_ line, regardless of whether the photon included is from fluorescence or bremsstrahlung emission. This has the advantage of including more photons in the reconstruction, improving the signal-to-noise ratio. However, it includes X-rays generated outside the Pt layer, degrading the spatial resolution. The amount of degradation depends on how many photons generated outside the Pt are included in the reconstruction and what their effective focal spot size is. We find that when fitting to the Pt L_*α*_ line, 600 million photons are included in the reconstruction. When binning from 9.1–10.1 keV and including the Pt L_*α*_ line, about 1 billion photons are included, increasing the photon counts by a factor of 1.67. In the 9.1–10.1 keV range, the PENELOPE models indicate that 87% of all detected photons originate from the Pt thin film. Therefore, a large proportion of the photons used still originate from the nanoscale X-ray spot generated in the Pt layer. In the reconstructions shown here, the coarser energy-binning approach was used rather than the fit to the Pt L_*α*_ line. It is unlikely that this coarse binning approach will continue to be effective as the spatial resolution of MINT is pushed to the sub-100 nm level in future experiments. Thinner targets are required to generate smaller spot sizes, which will stop fewer electrons. Thus, we can expect a higher proportion of the bremsstrahlung to originate from materials other than the target, which will have a larger negative impact on image quality.

The energy resolution of the coadded array was found to be 17.9 eV at the Cu K_*α*_ line (Figure 6C). The Cu K_*α*_ line was used to measure resolution because its line shape is well known [36,37]. This energy resolution is sufficient to separate the Pt L_*α*_ counts from the background with a high signal-to-noise ratio, but it does not represent the state of the art for TES pixels. TES pixels are capable of achieving much higher energy resolutions than what is demonstrated here, with resolving powers (E/ΔE) greater than 1000 [15]. The current TES array was optimized for faster photon detection to be compatible with the X-ray generation rate of the SEM at the cost of a minor degradation in the energy resolution [30].

### 3.2. Image Generation

Both two-dimensional radiographs and three-dimensional tomographic reconstructions were generated from the collected data. Details on the data collection can be found in the Supplementary Materials, Section S4. Due to a combination of positional drift and offset in the commanded and executed angular and linear positions of the stage, radiographs collected during each scan can be offset. The offset was found to be minor between inner and outer regions collected at the same angle but significant across different angular projections. If not accounted for, this misalignment would degrade image resolution in the three-dimensional reconstruction. Radiograph alignment software was developed to quantify and correct for these positional offsets. The software operates by first aligning the two-dimensional radiographs from a scan at a given projection angle. Then, angle-to-angle alignments are performed in a sequential fashion with adjacent angles aligned to one another (e.g., 0° to ±7.5°, ±7.5° to ±15°, etc.).

To generate a three-dimensional image from aligned two-dimensional radiographs, the code TomoScatt was used. TomoScatt utilizes an objective function with a Bayesian prior that evaluates the log-likelihood that a given reconstruction is optimal [40]. The code was edited to accommodate a finite source and aspects of the TES spectrometer, such as pixel positioning and energy-specific reconstructions. Self-absorption was accounted for in the reconstructions. The reconstruction was also adjusted for attenuation in the Si spacer layer such that the final reconstruction was blind to Si. Additional information regarding reconstruction algorithm development and results can be found in a recent publication [21]. In total, the reconstruction utilized 1,005,243,897 photons in the 9.1–10.1 keV bin and an additional 788,551,557 photons when the lower energy bin was included for element-sensitive reconstructions. This corresponds to a detected photon rate of 1113 and 1987 counts per second, respectively.

## 4. Reconstruction Results

**Figure 7:**
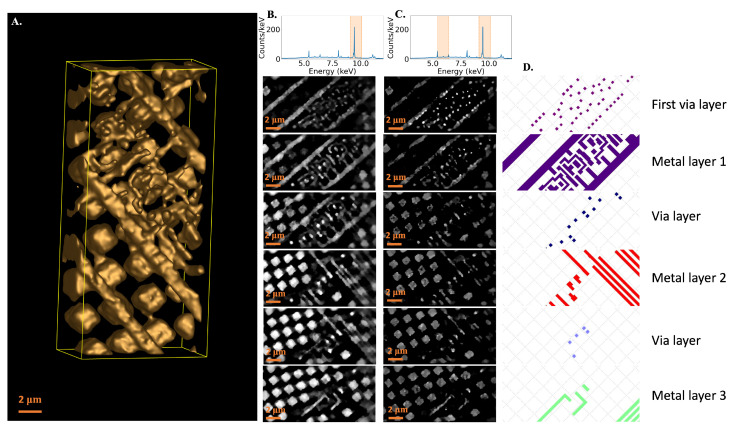
(**A**) Three-dimensional reconstruction of an IC fabricated at the 130 nm node, using X-rays in the 9.1–10.1 keV energy band. This band includes all Pt L_*α*_ photons. (**B**) Spectrum from the TES detector, with the 9.1–10.1 keV energy band used for reconstruction highlighted in orange (top). Reconstruction results, separated by the IC layer, are shown under the spectrum. These slices were taken from the reconstruction shown in A. (**C**) Multi-energy reconstruction results, using the 9.1–10.1 keV and the 5.4–6.4 keV band, shown under the TES spectrum, with the X-ray energies used highlighted in orange. Here, only the first via layer is resolved more clearly than when only using 9.1–10.1 keV photons, indicating a material other than Cu may be present. (**D**) GDS ground truth for each of the metal via and wiring layers, for comparison with the reconstruction results. A portion of this figure appeared in Ref. [21]. All scale bars in (**A**–**C**) are 2 μm wide.

The sample IC contains three metal wiring and three metal via layers with minimum feature sizes of 160 nm. Two reconstructions are shown: one using the 9.1–10.1 keV energy band, including the Pt L_*α*_ line (Figure 7A,B), and one using both the 9.1–10.1 keV band and the 5.4–6.4 keV band (Figure 7C). These reconstructions are compared to the wiring layers shown in the ground-truth graphic design system (GDS) file (Figure 7D), but the GDS file was not used as an input to either reconstruction. The larger cubes in the reconstructions represent chemical-mechanical polishing (CMP) fill. CMP fill is included in ICs during the fabrication process to provide thermal and mechanical stability [41]; it is not included in the GDS file.

Multiple reconstructions are shown to demonstrate the spatial resolution of MINT as well as the first steps toward element-sensitive reconstructions. The expected contrast for a given material changes based on the energy of photons used in the reconstruction (Supplementary Materials, Section S6). For Cu features, it is advantageous to use X-ray energies just above the Cu K-edge, making 9.1–10.1 keV an ideal choice. For other common IC materials, such as Ta or W, using lower energy bands is advantageous for improving contrast. However, the fraction of X-rays originating from the nanoscale spot in the Pt target layer decreases as lower energy bands are included. For the 5.4–6.4 keV band, only 51% of the detected photons are generated in the Pt target. Additionally, lower energy bands offer less contrast for Cu imaging than the 9.1–10.1 keV band. So, while including the lower energy band may improve contrast for non-Cu materials, it decreases contrast for Cu and leads to an increase in the X-ray focal spot size.

The first reconstruction, using only the 9.1–10.1 keV band, resolves all expected features in the IC. Wiring and via layers are clearly distinguished from the surrounding CMP fill. This is expected, as the majority of photons included in the reconstruction are generated in the Pt target and are at energies that provide a high Cu-SiO_2_ contrast. This demonstrates that MINT can resolve features at least as small as 160 nm. A small amount of crosstalk between layers is present due to the achievable spatial resolution in the thickness direction of the sample and the wiring and via layers being in physical contact with one another. While improvements in the spatial resolution of the instrument would improve this layer-to-layer crosstalk in the final reconstruction, the features in each layer are still clearly visible. The second reconstruction, which adds the 5.4–6.4 keV energy band into the reconstruction, is less effective at imaging most layers. However, it is more effective at resolving the first via layer, demonstrating higher contrast of the vias than the reconstruction using only the higher energy band. This suggests that the first layer is likely not comprised of Cu and is potentially W or Ta. Both W and Ta fluorescence peaks are visible in the TES spectrum, and both may be present in the IC. While future work is needed to fully explore the spectral imaging capabilities of MINT, the TES spectrometer offers several advantages for this imaging mode moving forward. The high resolving power of the TES allows many energy bands to be selected, offering the capability to identify many different materials simultaneously. Additionally, data across a broadband spectral range are collected in the same scan, so multiple reconstructions, including different energy bins, can be made from a single tomography dataset. These results demonstrate the capability of MINT in achieving nanoscale spatial resolutions and elemental sensitivity within a compact instrument and indicate the promise of utilizing SEM and TES-based systems for X-ray nanotomography.

## 5. Future Outlook

The current implementation of MINT is a proof of concept, demonstrating the potential of an SEM and TES-based approach to nanoscale tomography. The first generation of the tool, as shown here, achieves spatial resolutions comparable to state-of-the-art laboratory X-ray CT instruments, with additional capabilities for spectral imaging. MINT also has a clear path forward to further improve its spatial resolution, imaging speed, and spectral imaging capabilities. These upgrades involve improvements to the hardware, target design, and reconstruction approach but do not alter the overall MINT concept.

First, the electron column could be upgraded to achieve higher beam currents at smaller spot sizes, improving both the imaging speed and resolution. Modern electron columns can achieve microampere beam currents, significantly higher than what was used for the demonstration presented here [42,43]. Additionally, at a desired spatial resolution of 160 nm, the target can be designed such that the majority of X-rays are generated in the target layer. In this limit, a commercial megapixel camera could be used to drastically improve imaging speed at the cost of elemental-sensitive reconstructions. Using an example commercial X-ray detector (Dectris Eiger2), we estimate that the imaging speed could be approximately 850 times faster than the current 240-pixel TES spectrometer. However, at desired spatial resolutions below 100 nm, it would become difficult to maintain a thin enough target layer, small enough electron spot size, and sufficient electron beam current for imaging while keeping the majority of X-ray generation contained to the thin-film metal target. This would result in slow imaging speeds or a degradation in the achievable spatial resolution as the beam current is increased. One approach to push the spatial resolution to the level of feature sizes in state-of-the-art ICs is to isolate photons from one or multiple nanosized X-ray targets, which would contain the fluorescence X-ray emission from each material to a nanoscale spot size regardless of the size and current of the incident electron beam. Here, an energy-resolving detector would be essential for isolating the fluorescence from each nanotarget. A TES spectrometer could then be used to isolate X-ray emission from specific fluorescence lines of interest from multiple nanofeatured materials [44].

In addition to source upgrades to improve imaging speed and resolution, advances in TES detector technology will continue to improve energy resolution while drastically improving collection efficiency, enabling faster imaging speeds while maintaining elemental selectivity. These advances include individual TES pixels with higher count-rate capabilities and higher pixel-count TES spectrometers, which collect over a larger solid angle. Advances in TES multiplexing technology have already enabled the development of spectrometers with upwards of 1000 pixels, dramatically increasing photon collection capabilities in MINT [20]. If integrated into the current tool, this advancement would immediately improve imaging speed by a factor of 14. Lastly, the use of an energy-resolving detector creates the opportunity for spectral imaging approaches that could determine the elemental or chemical composition of the sample. A proof of concept for element-specific reconstructions with a TES was demonstrated here, but more advanced spectral tomography cases at synchrotron beamlines have resulted in the combination of tomographic and spectroscopic techniques to yield information on chemical states within each voxel [45,46,47]. TES spectrometers are widely used for X-ray spectroscopy [15,29] and have a history of bringing synchrotron techniques to the laboratory bench [48], creating the opportunity to develop advanced tabletop spectral tomography approaches centered around the MINT concept.

## 6. Conclusions

MINT represents an innovative approach to X-ray tomography, addressing many of the limitations of X-ray sources, system geometry, and X-ray detection that hinder conventional laboratory CT instruments. MINT combines a scanning electron microscope (SEM) with a transition-edge sensor (TES) spectrometer to enable X-ray tomographic imaging with nanoscale spatial resolutions and elemental specificity. The tightly focused electron beam spot generates X-rays on a metal target layer, maintaining a small focal spot size and eliminating the need for X-ray focusing optics. The broadband detection and energy resolution of the TES array are then used to select specific energy bands to emphasize spatial resolutions or the detection of different materials in the sample. A proof-of-concept measurement indicates that MINT can resolve features down to 160 nm in a planar Cu-SiO_2_ integrated circuit (IC) sample with a limited number of angular projections, with great potential for future improvement. MINT is inherently flexible, with the ability to adjust X-ray target materials, electron beam parameters, and geometric magnification to achieve optimal imaging conditions for a desired spatial resolution or sample composition. The current implementation of MINT demonstrates the promise of merging an SEM with energy-resolved X-ray detection for nanoscale tomography, and the next generation could achieve nanoscale, element-specific tomography not previously possible with laboratory X-ray CT instruments.

## Data Availability

The raw data supporting the conclusions of this article will be made available by the authors on request.

## Supplementary Materials

The following supporting information can be downloaded at https://www.mdpi.com/article/10.3390/s24092890/s1: Figure S1: SEM Surface Images; Figure S2: EDS Spectrum; Figure S3: Tomographic Scan Area; Figure S4: Sample Positioning System; Figure S5: Positional Drift Magnitude; Figure S6: Contrast of Common IC Materials. References [34,37,38,49,50,51,52,53,54,55] are cited in the Supplementary Materials.
