# Supplementary material for: Nanoscale Three-Dimensional Imaging of Integrated Circuits Using a Scanning Electron Microscope and Transition-Edge Sensor Spectrometer
Source: Sensors (Basel). 2024 Apr 30;24(9):2890. doi: 10.3390/s24092890 (PMC11086152; doi:10.3390/s24092890)
Supplement: Supplementary file 1 [file sensors-24-02890-s001.zip › sensors-2923346-supplementary.pdf]

Supplemental Material
Nanoscale Three-Dimensional Imaging of Integrated Circuits using a Scanning Electron Microscope and Transition-Edge Sensor Spectrometer

Nathan Nakamura[1,2a)], Paul Szypryt[1,2], Amber L. Dagel[3], Bradley K. Alpert[1], Douglas A. Bennett[1], William Bertrand Doriese[1], Malcolm Durkin[1,2], Joseph W. Fowler[1,2], Dylan T. Fox[3], Johnathon D. Gard[1,2], Ryan N. Goodner[3], James Zachariah Harris[3], Gene C. Hilton[1], Edward S. Jimenez[3], Burke L. Kernen[3], Kurt W. Larson[3], Zachary H. Levine[4], Daniel McArthur[3], Kelsey M. Morgan[1,2], Galen C. O'Neil[1], Nathan J. Ortiz[1,2], Christine G. Pappas[1,2], Carl D. Reintsema[1], Daniel R. Schmidt[1], Peter A. Schultz[3], Kyle R. Thompson[3], Joel N. Ullom[1,2], Leila Vale[1], Courtenay T. Vaughan[3], Christopher Walker[3], Joel C. Weber[1,2], Jason W. Wheeler[3], and Daniel S. Swetz[1]

[1]National Institute of Standards and Technology, Boulder, Colorado 80305, USA
[2]Department of Physics, University of Colorado, Boulder, Colorado 80309, USA
[3]Sandia National Laboratories, Albuquerque, New Mexico 87123, USA
[4]National Institute of Standards and Technology, Gaithersburg, Maryland 20899, USA
[a)]nathan.nakamura@nist.gov

S1 Imaging Speed

A derivation of Equation 1 is given here, describing how the relative imaging speed was determined. The N measured photons consist of B background bremsstrahlung photons plus F fluorescence photons in the peak, with

$$F = N - B. \tag{1}$$

As F is the difference between two independent Poisson random deviates, it follows the Skellam distribution[1], with a variance

$$\sigma^2(F) = E[N] + E[B] \approx F + 2B. \tag{2}$$

Where E[] represents the expectation value of the enclosed variable. We define f to be the average count rate of fluorescence photons, b to be the average bremsstrahlung count rate per eV, and t to be the integration time. Then, $E[F] = ft$, and the uncertainty on our estimation of F can be expressed as

$$\sigma(F) = \sqrt{ft + 2b\Delta Et} \tag{3}$$

where ΔE is the signal bandwidth in eV—a combination of the intrinsic emission-line width and the resolution of the TES spectrometer at the fluorescence line of interest.

The goal of transmission-based X-ray tomography is to distinguish different materials in each voxel by their X-ray transmission. The transmission is $T = \exp(-\mu x)$ through a voxel of thickness x given an X-ray absorption of μ per unit length. Considering only one voxel, if F_0 were the expected number of fluorescence photons that would be seen if that voxel had zero absorption ($\mu = 0$) and all other layers of voxels were unchanged, the expected value of F would be

$$E[F] = F_0 \exp(-\mu x). \tag{4}$$

To first order, the relationship between uncertainties on F and μ would be

$$\sigma(F) = xF\sigma(\mu). \tag{5}$$

We define $\Delta\mu$ as the intrinsic contrast between Cu and SiO_2. Thus, a measurement with $\sigma(\mu) < \Delta\mu/k$ will have a signal-to-noise ratio of k for the Cu/SiO_2 discrimination task. The requirement is that the absorption contrast meets the condition

$$\Delta\mu > k\sigma(\mu) = \frac{k\sigma(F)}{xF} \tag{6}$$

$$> \frac{k}{x}\frac{\sqrt{ft + 2b\Delta Et}}{ft} \tag{7}$$

$$> \frac{k}{x}\sqrt{\frac{1 + 2b\Delta E/f}{ft}}. \tag{8}$$

Rewriting in terms of imaging speed $1/t$, we get

$$\frac{1}{t} < f\left(\frac{x\Delta\mu}{k}\right)^2 (1 + 2b\Delta E/f)^{-1}. \tag{9}$$

The values of x and k are not relevant to the calculation of relative imaging speed given in the main text, and thus are omitted. When predicting the absolute imaging speed, the speed will scale as the square of the voxel thickness. Likewise, the time required grows as the square of the required per-voxel signal-to-noise.

S2 Sample Preparation

Preparation of the demonstration IC sample was required to achieve the desired system geometry for high magnification. The sample consists of Cu wiring in an SiO_2 dielectric on a Si substrate. First, the Cu/SiO_2 surface was polished (Allied Multiprep polisher) and surface topography was measured (Dektak profilometer, Bruker). Then, metal layers of the IC were removed via spin milling in an FEI G4 plasma focused ion beam (FIB) system. The metal layers removed from the IC contained larger Cu features and were not of interest for a nanotomography demonstration. Removing these larger wiring layers is not strictly necessary, but it enables faster imaging of the remaining layers. After layer removal, three metal wiring and three metal via layers remain. The Cu/SiO_2 surface was then mounted on a 60 μm thick glassy carbon layer (SPI Supplies) using Epotek 377 epoxy to provide structural rigidity. After sample preparation, the Cu/SiO_2 sample was approximately 3.5 μm thick. This resulted in approximately 10% absorption in the sample during data collection, depending on the effective sample thickness due to sample rotation.

The Si substrate on the backside of the IC was thinned to the desired thickness, in this case 8.5 μm. The thickness of the Si layer, referred to as the spacer layer, depends on the intended spatial resolution of the measurement (see Section II.B). To thin the spacer, the surface was lapped (Allied Multiprep) and polished, with the thickness verified using an Allied Vision System reflectometer. The glassy carbon-facing side of the sample is then bonded to a graphite puck using Epotek 377. The graphite puck serves as a sample carrier for further processing and insertion into the MINT sample holder. The puck has a window cut into the back aligned with the sample mounting position to reduce photon attenuation.

Once on the graphite puck, fiducial marks of tungsten are deposited on the Si surface using the FEI G4 plasma FIB (Fig. S1A). The fiducials are deposited around the scan region of interest (ROI) and are used both for macroscale localization of the

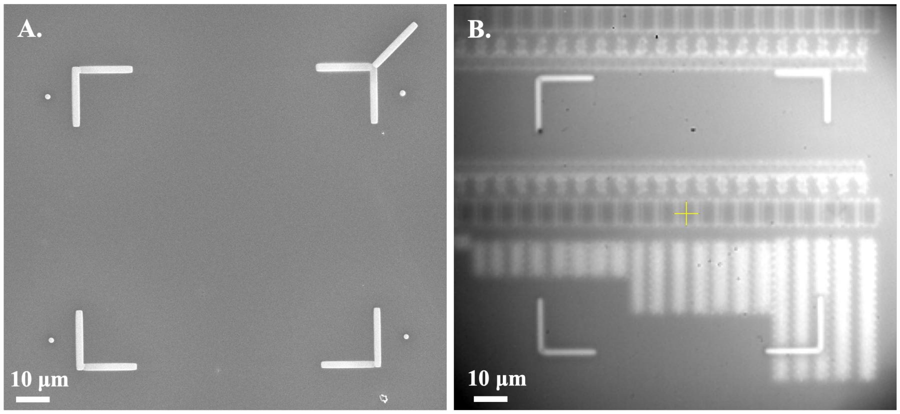

Figure S1. (A) SEM image of the Pt target and W fiducials (B) Infrared (IR) image of the sample surface, showing the W fiducial position relative to IC features. The tomographic region of interest is within the area enclosed by the W fiducials.

ROI and for positional corrections during tomographic data collections (Fig. S1B). The X-ray target is then deposited directly over these fiducials and onto the spacer layer. For the current IC sample, 10 nm of Cr was evaporated as an adhesion layer followed by 100 nm of Pt.

S3 Data Processing

A TES Data Processing

1 Pulse Processing

The steps for TES data processing are described in more detail here. First, a series of cuts are performed to remove anomalous pulses. The majority of pulses cut are excluded due to pulse pileup effects, in which a second pulse arrives during the thermal decay of a previous pulse. After irregular pulses are removed, an average pulse is created for each pixel and used along with the noise autocorrelation function to create an optimal filter that maximizes the signal-to-noise ratio (SNR) during pulse height estimation.[2,3]

The measured pulse height in each pixel can be affected by a number of other physical effects beyond the energy of the incident photon, namely temperature drift on the (unregulated) 1 K stage of the ADR and the arrival time of a photon relative to the digital sampling clock. We correct for temperature-dependent drift with an algorithm that sharpens the energy spectrum by minimizing spectral entropy. It uses the mean pretrigger period of each pulse record as an indicator of temperature drift; sensor gain is found to vary linearly with this drift. Various approaches can be take to mitigate the arrival-time bias; most importantly, we smooth the optimal filter by a 1-pole filter with a 3 dB point of approximately 5 kHz to reduce arrival-time sensitivity. Additional details on these biases and corrections can be found in a prior publication.[4]

The optimally filtered, temperature drift-corrected, and arrival time-corrected pulse heights are calibrated into energy using known fluorescence lines. Here, the Pt L_α, Pt L_β, Cu K_α, and Cr K_α lines are used as calibration anchor points. Each line is fit to a model consisting of a known line shape[5,6] and a cubic spline is used to create a gain function relating the pulse height to X-ray energy.[7] The end result of these processing steps is a series of time-lagged, energy-calibrated X-ray pulses, which can be used to generate an energy-calibrated spectrum.

2 TES Pixel Screening

Prior to including data in the tomographic reconstruction, each pixel is screened to ensure that it is operating as intended. Each TES is screened based on the number of cut pulses, the Pt L_α count rate, and the energy resolution at the Pt L_α line. A TES pixel is flagged and excluded from the dataset if more than 20 % of detected pulses were cut, if the Pt L_α count rate is more than 25 % higher or lower than the median rate across all pixels, or if the energy resolution at the Pt L_α line is worse than 60 eV (FWHM). This eliminates pixels with manufacturing defects or errors in TES configuration that cause irregular pulse shapes or poor energy resolution. On average, 193 of the TES pixels were used in a given scan. In the TES pixels that passed these checks, 94 % of X-ray pulses were kept and used for subsequent analysis steps.

B EDS Data Processing and Analysis

The energy dispersive spectroscopy (EDS) detector is used as a monitor of fluctuations in the X-ray source term to normalize the detected transmission signal from the TES spectrometer. The X-ray source intensity can vary both spatially and temporally due to variations in the target thickness and SEM beam current. During MINT data collection, the EDS data is saved every second and consists of the detected counts per 10 eV bin, the timestamps at the beginning and end of each collection, and the detector's effective live time. Due to the high count rates incident on the EDS detector, pulse pileup occurs which renders certain subsets of the EDS data unusable by the Aztec Software system. The effective live time is the portion of the dwell not affected by pulse pileup. Here, the typical EDS live time was 0.75 seconds per 1 second dwell. The EDS timestamps are used to align the EDS spectra with the corresponding tomographic dwell and its TES and positional data. Then, all EDS spectra contained in a given dwell are summed to generate a final spectrum (Fig. S2). The final EDS spectrum for a given dwell has an energy resolution of approximately 200 eV at the Pt L_α line and tracks the source intensity at each tomographic dwell position. The Pt L_α count rate was used for X-ray source term normalization during tomographic reconstructions, as it encompasses both fluctuations in the SEM beam current and variations in the target thickness at each dwell position. The Pt L_α count rates were found by fitting to the fluorescence line for each dwell and dividing by the live-time corrected dwell time.

C Positional Data

Achieving nanoscale spatial resolution requires similarly precise knowledge of the source position on the sample. During each tomographic scan, the x, y, and z stage positions are stored relative to the IC sample plane-of-reference for both the beginning and end of the dwell period. This provides known electron beam positions for the start and end of the dwell, allowing the magnitude of the drift during each dwell period to be easily calculated. Additionally, the rotation angle of the stage is saved at each dwell.

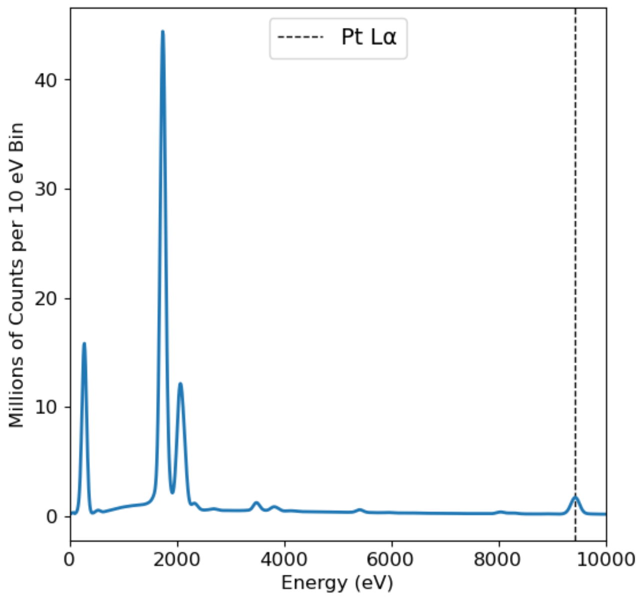

Figure S2. Spectrum collected by the EDS detector summed over all dwell positions. The Pt L_α line (vertical black dashed line) is fit and used to estimate fluctuations in the X-ray source during data collection. These fluctuations can arise from small variations in the Pt layer thickness over the scan region or fluctuations in the electron beam current.

D Data Consolidation

MINT requires the collection of data from multiple sources, namely the stage, SEM, TES spectrometer, and EDS detector. To facilitate their use in tomographic reconstruction codes, it is advantageous to consolidate the data into one input file. MINT data products relevant to tomography were stored in a Hierarchical Data Format version 5 (HDF5) file, with one HDF5 file per inner or outer scan. These HDF5 files include the TES Pt L_α and total count rates per pixel per dwell, the EDS total and Pt L_α count rates per dwell, the stage positions at the beginning and end of each dwell, the TES pixel positions relative to the center of the TES array, and timestamps for the beginning and end of each dwell, along with various other TES diagnostics not used for tomographic reconstructions. In total, the tomographic reconstruction codes utilized 74 HDF5 files over the angular range with 18 outer region scans and 56 inner region scans.

S4 Scan Strategy and Implementation

A scan plan for tomographic data collection was developed based on MINT system parameters. The ADR hold time at 100 mK limited the data acquisition time per day to approximately 18 hours. The scan area, step sizes, and dwell time at each step were chosen based on this time allotment. To maximize the amount of time spent collecting data during the ADR hold time, one angular projection was collected each day and setup for a new angular projection was performed during the ADR magnet cycle, which takes approximately four hours to complete. Data at each angle was collected over a series of scans of a smaller inner region and a larger outer region, with the feature-dense digital logic region approximately centered in each (Fig. S3). Three 11 μm $\times$ 4 μm inner region scans were collected to provide high spatial resolution in the digital logic region. In the inner region scans, a step size of 405 nm was used, with a dwell time of 45 seconds per dwell. The exact dwell positions between each inner region were offset slightly to provide a denser coverage of the region-of-interest (ROI) across all three inner region scans. After the inner region scans were complete, one 16 μm $\times$ 5 μm outer region scan was collected at a step size of 700 nm and dwell time of 65 seconds. The outer region is collected to mitigate edge effects in the inner region scans, which degrade the spatial resolution on the outer edges of the ROI.[8,9] As the sample rotates to higher angles around the y-axis, the projection of the ROI extends in the x-axis. This leads to a larger ROI at higher angles. To complete all four scans within the ADR hold time and maintain the same dwell density within the rotated scan regions, the step sizes and dwell times were adjusted from the values given above.

During a scan, corrections must be made periodically to account for positional drift of the electron beam or stage to ensure that the data is collected at the desired dwell location. To accomplish this, SEM images of a W fiducial at the same commanded stage location are taken before and after the first dwell position and after each subsequent dwell. Each post-dwell image is compared and aligned to the pre-dwell image, with the amount of alignment needed corresponding to the amount of positional

drift which occurred during that dwell. Any positional offsets are integrated into the next dwell position to correct for drift. This process of fiducial alignment and positional corrections adds approximately 20 seconds of overhead per dwell. Details on the sample positioning system and positional drift during scanning can be found in Sections S5-S6.

Data were collected over a total of 12 angles, ranging from $-37.5°$ to $+45°$ in $7.5°$ angular steps. The angular step is set by the angular coverage provided by the x-ray detector. In MINT, a spectrum is collected at each dwell position in the scan region. A reconstruction using the collected spectra at a single projection angle is equivalent to $7.5°$ of angular coverage. The total angular range is set by limitations in the sample and system geometry. In general, the angle of rotation is limited for planar samples due to the increasing path length to exit the sample at higher rotation angles. This is a challenge facing CT scans on planar samples in all tomography systems, as the increased attenuation at higher path lengths through the sample can drastically reduce the number of transmitted photons available for image generation. In MINT, two additional challenges for planar samples arise due to the unique system configuration. First, a risk of stage collision with the SEM column exists at angles larger than $\pm 45°$. Second, at angles more negative than $-37.5°$ the sample holder obscures the EDS detector and the source term cannot be estimated. The angular step size was selected based on the solid angle of the TES array, which provides $7.5°$ of angular coverage.

S5 Stage and Sample Holder

To perform tomography, a sample positioning system (SPS) which enables high-precision *x*, *y*, *z*, and angular motion is required. The MINT SPS consists of three commercial stages (Physik Instrumente) mounted in series, with associated positional sensors.[10] The system provides a total of 13 actuated degrees of freedom (DOF), of which seven are regularly used during tomographic data collection. For larger scale (cm) motion, a 6-DOF hexapod stage is mounted at the base of the SPS. A high-precision 6-axis flexure stage is mounted atop the hexapod and provides positional accuracy of a few nm across a translational range of 200 μm. The flexure stage utilizes piezoelectric actuators mounted to flexure bearings, eliminating backlash associated with screws, gears, and roller bearings. A single-axis rotation stage is mounted atop the flexure stage and utilizes a ratcheting piezoelectric actuator with an incremental encoder. Each stage contains internal sensors for positional control, while external interferometric and capacitative distance sensors provide additional, global position sensing capabilities. The entire SPS is shown in Fig. S4A.

A custom sample holder assembly is attached to the output of the rotation stage (Fig. S4B). The graphite puck with the mounted IC is placed into a circular recess on the sample holder and held in place with a face plate on the front side and spring-loaded brass clips on the back. The entire sample holder is locked on the sample receiver base by a spring-loaded locking mechanism. The mechanism consists of a hook attached to the sample receiver base which rotates downward against a rod attached to the underside of the sample holder, securing the sample holder against the sample receiver base (Fig. S4C).

S6 Positional Drift

During data collection, the electron beam dwells at a specific scan location on the target layer for 45 to 60 seconds, depending on the scan being collected and the rotation angle. The positional drift of the electron beam on the target surface and of the sample

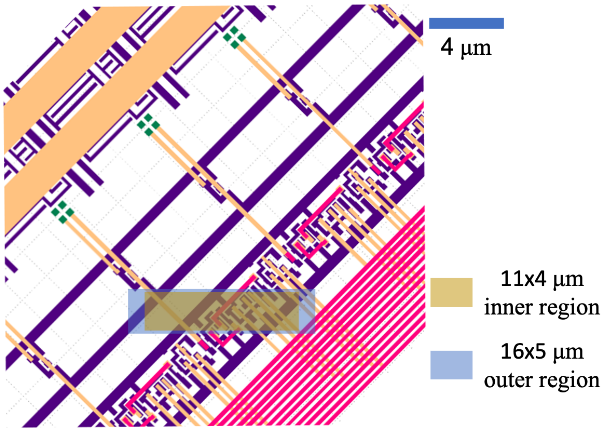

Figure S3. Metal layers of the demonstration IC sample. The sample was thinned to contain three metal (yellow, purple, pink) and three via layers (not shown). The green features extend through multiple metal and via layers. The inner (yellow) and outer (blue) rectangular scan regions were chosen to cover the digital logic region of the IC, which contains the densest population of 160 nm Cu features.

stage during this dwell period impacts the achievable spatial resolution by effectively blurring the spot size by a magnitude proportional to the amount drifted. Therefore, the position of the electron beam and stage must remain stable to within the resolution goal of the measurement, with any positional drift resulting in blurring of the final tomographic reconstruction. In addition to instabilities inherent to the stage and electron column, interference from ambient magnetic fields can cause instability in the electron beam. This can lead to unwanted noise in SEM images and poor positional control during tomographic data collection. An electromagnetic interference (EMI) cancellation system (Spicer Consulting) is used to mitigate the effects of ambient fields around MINT.

The W fiducials were used to characterize the positional stability of the electron beam and sample stage. During a drift characterization test, an SEM image of the W fiducial is taken prior to any stage movement. The flexure stage is then moved to the intended dwell location for one minute. After the dwell period, the flexure stage is moved back to the previous W fiducial position and another SEM image is acquired. The images are compared and aligned, with the movement needed to realign the fiducial in each image corresponding to the amount of positional drift during the dwell. This process is repeated for a number

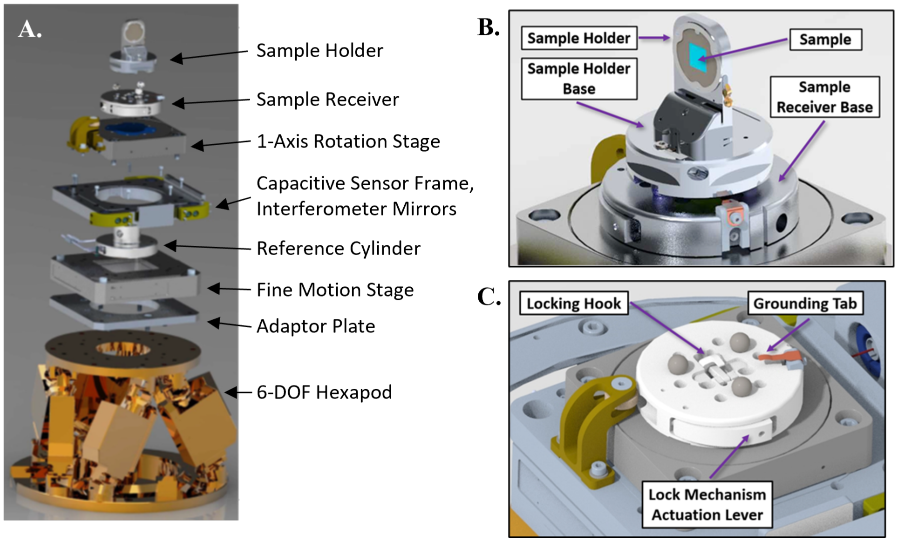

Figure S4. (A) The sample positioning stack, consisting of a three-stage sample positioning system (SPS), external capacitive and interferometric sensors, and custom-made sample holder and receiver. (B) The sample holder assembly and sample, attached to the sample receiver base. (C) The sample receiver base, which locks the sample holder securely to the SPS during tomographic data collection.

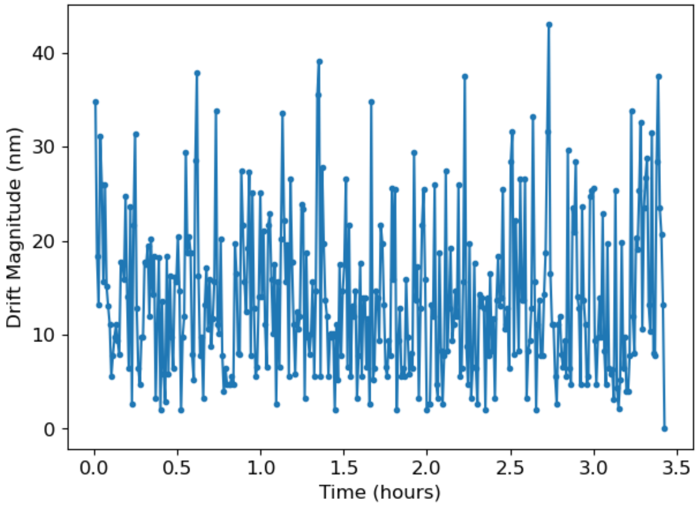

Figure S5. Positional drift magnitude per minute over a 3.5 hour period. The positional stability of the SEM beam and stage is within the limit needed to perform nanoscale tomography.

of dwells, with each post-dwell fiducial image being compared to the fiducial image taken after the dwell prior. Fig. S5 shows a plot of the positional drift magnitude per minute over a 3.5 hour period. Over all data sets used for tomographic reconstruction, the mean drift per dwell was 15 nm, at dwell times of approximately 45 seconds.

S7 Contrast

We defined the contrast between Cu and SiO_2 as the difference in attenuation $\Delta\mu$. The contrast is a function of the incident photon energy and the material being imaged, and its value at a certain energy is representative of how well photons at that energy differentiate Cu features from SiO_2. This calculation can be performed for any candidate material, and can serve as an indicator for which energy bands will be most useful for reconstructions of a given material. Fig. S6 shows $\Delta\mu$ for common IC materials.

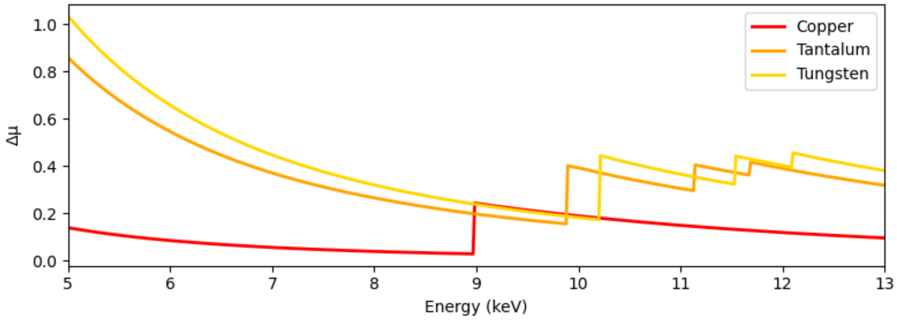

Figure S6. $\Delta\mu$ between a given material and SiO_2 for Cu, Ta, and W. Cu is the predominant wiring and via material in the sample IC, while Ta and W are candidate materials for the first via layer. The first via layer becomes significantly clearer when including the 5.4-6.4 keV band, indicating a non-Cu material may be present.

References

[1] J. G. Skellam, "The frequency distribution of the difference between two poisson variates belonging to different populations", J. Royal Statistical Society **109**, 296 (1946).

[2] A. E. Szymkowiak, R. L. Kelley, S. H. Moseley, and C. K. Stahle, "Signal processing for microcalorimeters", en, Journal of Low Temperature Physics **93**, 281–285 (1993).

[3] B. Anderson and J. Moore, *Optimal filtering*, Information and system sciences series (Prentice-Hall, 1979).

[4] J. W. Fowler, B. K. Alpert, W. B. Doriese, Y. I. Joe, G. C. O'Neil, J. N. Ullom, and D. S. Swetz, "The practice of pulse processing", Journal of Low Temperature Physics **184**, 374–381 (2016).

[5] G. H. Zschornack, *Handbook of x-ray data* (Springer, 2007), p. 969.

[6] G. Hölzer, M. Fritsch, M. Deutsch, J. Härtwig, and E. Förster, "$K\alpha_{1,2}$ and $K\beta_{1,3}$ x-ray emission lines of the $3d$ transition metals", Phys. Rev. A **56**, 4554 (1997).

[7] J. W. Fowler, B. K. Alpert, G. C. O'Neil, D. S. Swetz, and J. N. Ullom, "Energy calibration of nonlinear microcalorimeters with uncertainty estimates from gaussian process regression", Journal of Low Temperature Physics **209**, 1047–1054 (2022).

[8] A. Kyrieleis, V. Titarenko, M. Ibison, T. Connolley, and P. Withers, "Region-of-interest tomography using filtered backprojection: assessing the practical limits", Journal of Microscopy **241**, 69–82 (2011).

[9] R. N. Chityala, K. R. Hoffmann, D. R. Bednarek, and S. Rudin, "Region of interest (ROI) computed tomography", in Medical Imaging 2004: Physics of Medical Imaging, Vol. 5368, edited by M. J. Yaffe and M. J. Flynn (International Society for Optics and Photonics, 2004), pp. 534–541.

[10] C. L. Willis, E. M. Lavely, A. J. Marcinuk, P. R. Moffitt, and J. R. Takahashi, *Sample manipulation for nondestructive sample imaging*, US Patent 10535495, 2020.
